# Nicotinamide Ameliorates Deoxynivalenol-Induced Injury in Renal Cells via Inhibiting PARP1 Hyperactivation and Restoring NAD^+^ Homeostasis

**DOI:** 10.3390/toxins18050227

**Published:** 2026-05-10

**Authors:** Chao Chen, Yifan Qin, Zijun Luo, Peiqiang Mu, Jikai Wen, Yiqun Deng

**Affiliations:** 1 State Key Laboratory of Swine and Poultry Breeding Industry, College of Life Sciences, South China Agricultural University, Guangzhou 510642, China; cchen@scau.edu.cn (C.C.); qyf20020219@163.com (Y.Q.); luozijun1998@163.com (Z.L.); mpeiqiang@scau.edu.cn (P.M.); 2 Guangdong Academy of Agricultural Sciences, Guangzhou 510640, China

**Keywords:** deoxynivalenol, NAD^+^ homeostasis, PARP1, nicotinamide, renal cell injury, mitigation strategy

## Abstract

Deoxynivalenol (DON) is a globally prevalent mycotoxin that threatens food and feed safety via severe multi-organ toxicity. Previous studies indicate that DON induces cellular energy metabolism dysregulation by triggering oxidative stress and impairing mitochondrial function. During this process, nicotinamide adenine dinucleotide (NAD^+^), a central coenzyme in cellular energy metabolism, frequently exhibits significantly decreased intracellular levels or even complete depletion. However, the molecular mechanisms underlying the disruption of NAD^+^ homeostasis by DON exposure, as well as the development of targeted countermeasures, remain elusive. Using human embryonic kidney 293T (HEK293T) cells as an in vitro renal toxicity model, we dissected DON-induced NAD^+^ dysregulation and evaluated the protective potential of nicotinamide (NAM). DON caused significant NAD^+^ depletion in porcine serum (in vivo) and HEK293T cells (in vitro), which was confirmed as a key driver of cytotoxicity. Mechanistically, although DON binds and inhibits nicotinamide phosphoribosyltransferase (NAMPT), the rate-limiting enzyme of the NAD^+^ salvage pathway, neither NAMPT knockdown and overexpression nor nicotinamide mononucleotide (NMN) supplementation rescued DON-induced toxicity. Instead, DON dose-dependently activated poly(ADP-ribose) polymerase 1 (PARP1), the primary intracellular NAD^+^-consuming enzyme, to accelerate NAD^+^ depletion. PARP1 knockdown markedly attenuated DON-induced cytotoxicity, identifying PARP1 hyperactivation as the core toxic mechanism. NAM dose-dependently suppressed PARP1 activity, replenished NAD^+^ pools, and reversed cell injury. These findings establish PARP1-driven NAD^+^ depletion as an important mechanism of DON-induced renal toxicity, providing a promising intervention candidate for mitigating DON toxicity in food safety.

## 1. Introduction

Deoxynivalenol (DON), also referred to as vomitoxin, is a secondary metabolite synthesized by *Fusarium* fungi and is widely identified as a contaminant in cereal-based food and feed products across the globe [1]. According to the 2024 Cargill Global Mycotoxin Survey, DON has the highest global detection rate (up to 79%) among all conventional mycotoxins, far exceeding that of other co-occurring mycotoxins [2]. This toxin predominantly contaminates forage (including silage and hay), maize, wheat, barley, and their processed by-products, with particularly high contamination levels in major grain-producing regions across East Asia, North and Central America, and Europe [3,4]. The global prevalence of DON contamination poses a substantial threat to food security, livestock and poultry production sustainability, and public and animal health worldwide [4,5,6,7].

DON exerts multi-organ toxicity in animals, with well-documented damaging effects on the intestine, liver, kidney, and immune system [8,9,10,11]. As the primary excretory organ of the body, the kidney mediates the filtration and elimination of endogenous metabolic wastes and exogenous toxins (including DON), making it highly vulnerable to damage caused by toxin accumulation [12,13]. Previous studies have confirmed that DON exposure triggers inflammatory cell infiltration, hemorrhage, renal tubular degeneration, and glomerular atrophy in the murine kidney, further impairing renal function [12,14].

At the cellular level, DON binds to the 60S subunit of eukaryotic ribosomes to inhibit protein synthesis, disrupt mRNA translation, and trigger ribotoxic stress, which ultimately induces apoptotic cell death [15,16,17]. Meanwhile, DON exposure markedly promotes excessive intracellular reactive oxygen species (ROS) generation, leading to oxidative stress [18,19]. DON also activates the endoplasmic reticulum stress-related unfolded protein response (UPR) pathway, initiating apoptotic cascades via downstream molecules including CHOP and JNK, which are key mediators of DON-driven renal injury [20,21]. Notably, DON-induced oxidative stress and DNA damage can further activate intracellular NAD^+^-consuming enzymes, especially poly(ADP-ribose) polymerase 1 (PARP1), resulting in the disruption of intracellular NAD^+^ homeostasis [22,23].

To date, no specific antidote exists for DON intoxication. Existing studies have mainly focused on mitigating DON-induced systemic injury via modulation of the gut microbiota [14,24,25]. Recent studies have shown that polyphenolic compounds such as phlorizin, hesperidin and taxifolin effectively attenuate DON-induced damage. However, these investigations have largely focused on DON-induced intestinal injury, while the molecular mechanisms underlying DON-triggered renal damage (especially those related to NAD^+^ homeostasis dysregulation) and corresponding targeted protective strategies remain poorly characterized [25,26,27,28,29].

NAD^+^ is an essential intracellular coenzyme involved in multiple core biological processes, including energy metabolism and DNA repair. The maintenance of its intracellular homeostasis is indispensable for cellular function [22,30,31,32]. Nicotinamide phosphoribosyltransferase (NAMPT), the rate-limiting enzyme in the NAD^+^ salvage synthesis pathway, catalyzes the conversion of nicotinamide (NAM) to nicotinamide mononucleotide (NMN), which is further converted to NAD^+^ by nicotinamide mononucleotide adenylyltransferase (NMNAT), thus playing a pivotal role in regulating intracellular NAD^+^ homeostasis [33,34]. As the primary precursor of NAD^+^, NAM exerts multiple key biological functions [34]. It inhibits the NF-κB signaling pathway and downregulates the expression of pro-inflammatory cytokines including interleukin-6 (IL-6) and tumor necrosis factor-α (TNF-α), thereby suppressing excessive inflammatory responses [35]. NAM also increases intracellular NAD^+^ levels, enhances cellular antioxidant capacity, preserves normal mitochondrial metabolism, reduces mitochondrial ROS generation, and effectively attenuates DNA damage caused by oxidative stress [36]. In addition, NAM acts as an endogenous inhibitor of PARP1 by occupying its NAD^+^-binding pocket, suppressing its catalytic activity and limiting excessive NAD^+^ consumption [37,38]. Although direct experimental evidence remains lacking, the anti-inflammatory/antioxidant properties and central role of NAM in cellular energy metabolism theoretically endow it with the potential to mitigate the multiple toxic effects of DON.

In this study, we employed human embryonic kidney 293T (HEK293T) cells as an in vitro model to investigate the impact of DON on intracellular NAD^+^ homeostasis and its underlying molecular mechanisms. We specifically verified whether DON disrupts NAD^+^ homeostasis via inhibiting the NAMPT-mediated NAD^+^ synthesis pathway or activating the PARP1-mediated NAD^+^ consumption pathway, and evaluated the protective effect of NAM against DON-induced cellular injury. This study not only delineates the core mechanism of DON-induced renal injury from the perspective of NAD^+^ metabolism, but also provides solid in vitro experimental evidence for the development of NAM as a targeted detoxification strategy against DON toxicity.

## 2. Results

### 2.1. DON Exposure Triggers NAD^+^ Depletion In Vitro and In Vivo

**Figure 1:**
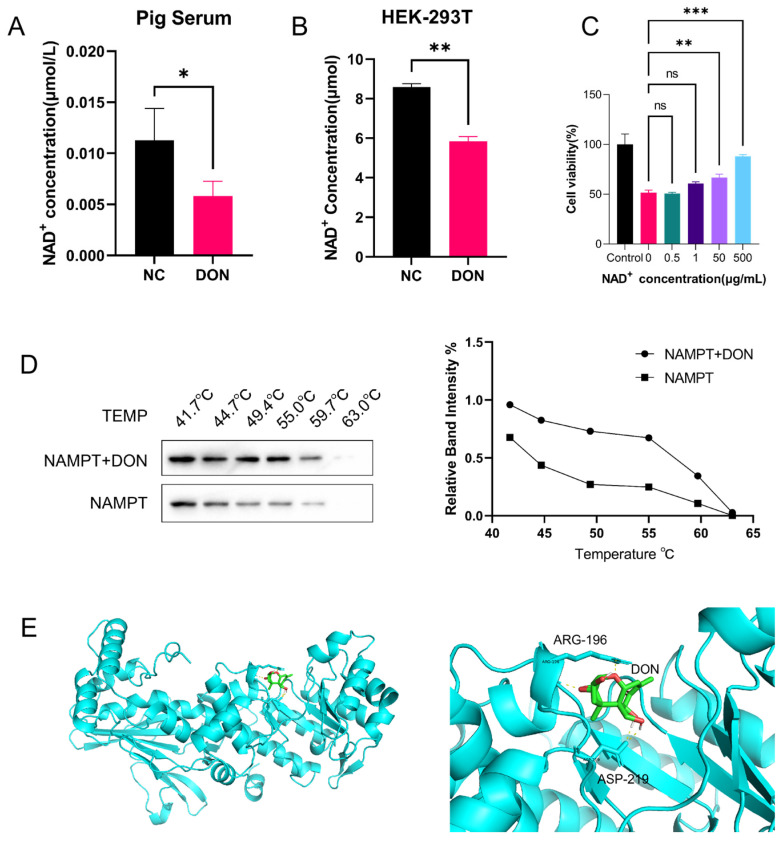
(**A**) Serum NAD^+^ content in Duroc × Landrace × Yorkshire three-way crossbred piglets after 28 days of DON challenge (2 mg/kg diet), *n* = 6 per group. (**B**) Intracellular NAD^+^ levels in HEK293T cells treated with 200 ng/mL DON for 24 h. (**C**) Protective effect of exogenous NAD^+^ (0, 0.5, 1, 50, 500 μg/mL) on DON-induced reduction in HEK293T cell viability (200 ng/mL DON for 24 h), detected by CCK-8 assay. (**D**) Purified recombinant human NAMPT was incubated with 100 μM DON or equal-volume DMSO (vehicle control) at 4 °C for 1.5 h. Aliquots were heated at the indicated temperature gradient for 3 min, and centrifuged to remove precipitated denatured proteins. Soluble NAMPT in supernatants was analyzed by Western blot. The line graph shows the normalized grayscale analysis with unheated protein serving as the internal standard. (**E**) Molecular docking model of DON and human NAMPT (PDB ID: 4KFN) indicates the key binding sites (Arg-196 and Asp-219) in the NAMPT active site. Statistical significance was designated as * *p* < 0.05, ** *p* < 0.01, and *** *p* < 0.001; ns = no significant difference.

We first assessed the impact of DON exposure on systemic and intracellular NAD^+^ levels in vivo and in vitro. NAD^+^ content was significantly reduced in the serum of DON-challenged pigs (Figure 1A) and in DON-treated HEK293T cells (Figure 1B), demonstrating that DON exposure directly disrupts NAD^+^ homeostasis at both the systemic and cellular levels. To establish a causal link between NAD^+^ depletion and DON-induced cytotoxicity, we performed CCK-8 assays to evaluate the viability of HEK293T cells following exogenous NAD^+^ supplementation. As shown in Figure 1C, exogenous NAD^+^ significantly mitigated the DON-induced reduction in cell viability in a concentration-dependent manner, confirming that intracellular NAD^+^ depletion is a key driver of DON-mediated cytotoxicity.

To explore whether DON alters NAD^+^ levels by binding to proteins involved in the NAD^+^ synthesis pathway, we first screened for DON-interacting proteins via protein microarray. After incubation of Cyanine 3-labeled DON with the microarray, we detected a strong binding affinity between NAMPT and DON (Table A1). To further validate their direct interaction, we performed an in vitro thermal shift assay (TSA) in temperature melt mode using purified recombinant human NAMPT protein. The results (Figure 1D) showed that the thermal stability of recombinant NAMPT protein at high temperatures was significantly higher in the DON-treated group than in the control group, confirming the specific binding between NAMPT and DON in vitro [39].

Further molecular docking simulation (Figure 1E) revealed that the primary binding sites of DON on NAMPT are arginine-196 (Arg-196) and aspartic acid-219 (Asp-219), which are located in the active site of NAMPT responsible for binding its substrates NAM and 5-phosphoribosyl-1-pyrophosphate (PRPP) [40]. This finding suggests that DON competitively binds to the active site of NAMPT, potentially blocking substrate access and thereby inhibiting its enzymatic function. Taken together, we hypothesized that DON may reduce intracellular NAD^+^ levels by inhibiting NAMPT enzymatic activity, thereby leading to impaired cell viability.

### 2.2. DON Regulates NAMPT Expression in a Concentration-Dependent Manner, Which Does Not Mediate DON-Induced Cytotoxicity

**Figure 2:**
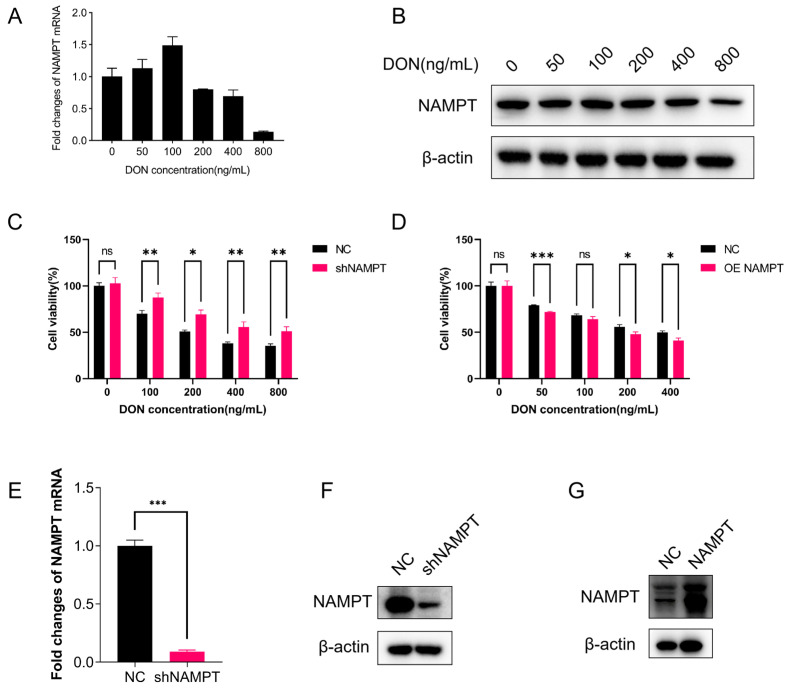
(**A**) *NAMPT* mRNA expression in HEK293T cells treated with DON (0, 50, 100, 200, 400, 800 ng/mL) for 24 h, detected by qRT-PCR. (**B**) NAMPT protein expression in DON-treated HEK293T cells, analyzed by Western blot (β-actin as loading control). (**C**) Cell viability of *NAMPT* knockdown (sh*NAMPT*) and negative control (shNC) HEK293T cells after gradient concentration of DON treatment for 24 h, detected by CCK-8 assay. (**D**) Cell viability of *NAMPT* overexpression (OE-*NAMPT*) and matching empty expression vector control HEK293T cells after gradient concentration of DON treatment for 24 h, detected by CCK-8 assay. (**E**) Knockdown of *NAMPT* was confirmed by qRT-PCR. (**F**) Knockdown of *NAMPT* was confirmed by Western blot. Matching empty shRNA vector was used as the negative control (NC). (**G**) Overexpression of *NAMPT* was confirmed by Western blot. Matching empty expression vector was used as the negative control (NC). Statistical significance was designated as * *p* < 0.05, ** *p* < 0.01, and *** *p* < 0.001; ns = no significant difference.

To investigate whether DON alters intracellular NAMPT abundance, HEK293T cells were treated with a gradient of DON concentrations for 24 h, and changes in *NAMPT* mRNA and protein expression were detected by quantitative real-time PCR (qRT-PCR) and Western blot, respectively. The qRT-PCR results (Figure 2A) showed that low concentrations of DON (50–100 ng/mL) significantly upregulated *NAMPT* mRNA levels, whereas high concentrations of DON (≥200 ng/mL) significantly downregulated its expression. Western blot results were consistent with the qRT-PCR findings (Figure 2B): NAMPT protein expression showed a trend of initial increase followed by a decrease with increasing DON concentration. These results indicate that DON regulates NAMPT expression in a concentration-dependent manner: it upregulates NAMPT expression at low concentrations and downregulates its expression at high concentrations.

To clarify the association between changes in NAMPT expression and DON-induced cytotoxicity, we evaluated the effect of NAMPT expression on cell viability via CCK-8 assay. We generated *NAMPT* knockdown (sh*NAMPT*) and *NAMPT* overexpression (OE-*NAMPT)* HEK293T cell lines (Figure 2E–G), which were treated with a gradient of DON concentrations for 24 h, respectively. The results (Figure 2C) showed that at all tested DON concentrations, cell viability in the sh*NAMPT* group was significantly higher than that in the negative control (shNC group), indicating that *NAMPT* knockdown significantly alleviated DON-induced reductions in cell viability. In contrast, *NAMPT* overexpression slightly increased the sensitivity of HEK293T cells to DON (Figure 2D), with marginally lower cell viability in the OE-*NAMPT* group than in the vector control group. These results were inconsistent with our initial hypothesis, indicating that although DON could regulate intracellular NAMPT protein levels, alterations in *NAMPT* expression were not the key mediators of DON-induced cytotoxicity.

### 2.3. DON Inhibits NAMPT Enzymatic Activity In Vitro, but This Is Not the Main Driver of DON-Induced Cytotoxicity

**Figure 3:**
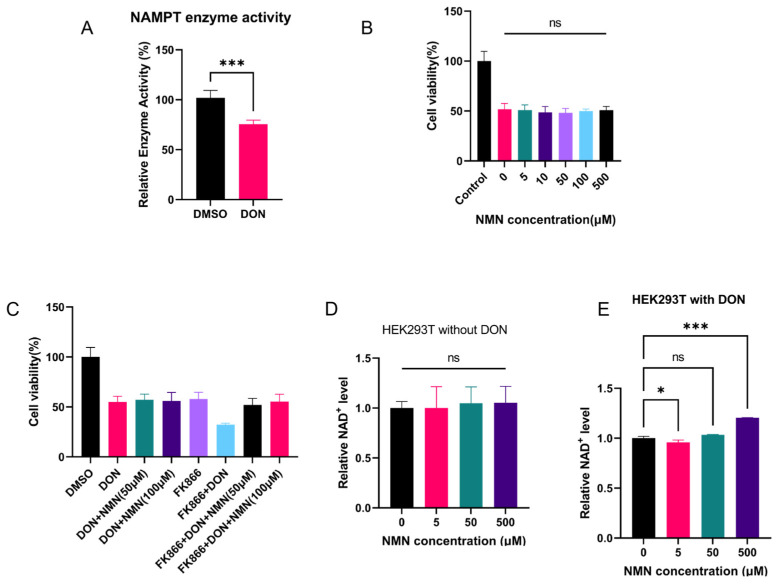
(**A**) In vitro enzymatic activity of recombinant NAMPT protein incubated with DON (3 μM), measured by fluorescent NMN derivative-based assay. (**B**) Effect of exogenous NMN (0, 5, 10, 50, 100, 500 μM) on the viability of HEK293T cells treated with DON (200 ng/mL) for 24 h. (**C**) Cell viability of HEK293T cells after single/combination treatment with DON (200 ng/mL), FK866 (1 nM), and NMN (50, 100 μM) for 24 h. (**D**) Intracellular NAD^+^ levels in untreated HEK293T cells supplemented with gradient concentrations of NMN (0, 5, 50, 500 μM). (**E**) Intracellular NAD^+^ levels in DON-treated HEK293T cells supplemented with gradient concentrations of NMN (0, 5, 50, 500 μM). Statistical significance was designated as * *p* < 0.05, and *** *p* < 0.001; ns = no significant difference.

We performed in vitro enzyme kinetic assays of recombinant NAMPT protein using a fluorescent NMN derivative-based quantitative method to investigate the effect of DON on NAMPT enzymatic activity. The results (Figure 3A) showed that the in vitro enzymatic activity of NAMPT was significantly lower in the DON-treated group than in the control group, indicating that DON directly inhibits the enzymatic activity of NAMPT in vitro.

Nicotinamide mononucleotide (NMN), the direct precursor of NAD^+^, can replenish intracellular NAD^+^ pools bypassing NAMPT [41]. To verify whether inhibition of NAMPT enzymatic activity is the main cause of DON-induced cytotoxicity, HEK293T cells were treated with DON for 24 h with exogenous supplementation of gradient concentrations of NMN, and cell viability was measured by the CCK-8 assay. The results (Figure 3B) showed that even with exogenous NMN supplementation, there was no significant alleviation of DON-induced cytotoxicity, and no significant difference in cell viability was observed among all treatment groups. This finding indicates that DON does not induce cellular damage by inhibiting NAMPT enzymatic activity to reduce NAD^+^ synthesis.

To further validate this hypothesis, we performed experiments using FK866 (N-[4-(1-benzoyl-4-piperidinyl)butyl]-3-(3-pyridinyl)-2E-propenamide), a specific NAMPT inhibitor [42]. FK866 effectively inhibits NAMPT enzymatic activity, while NMN can directly replenish intracellular NAD^+^ bypassing NAMPT and alleviate FK866-induced cytotoxicity [43]. We established the following groups: DMSO solvent control group, DON-alone treatment group, FK866-alone treatment group, DON and FK866 combined treatment group, and intervention groups supplemented with NMN (50, 100 μM) in each of the above treatment groups. Cell viability was detected after 24 h of treatment. The results (Figure 3C) showed that FK866 and DON alone induced a similar degree of cell viability inhibition, whereas combined treatment with DON and FK866 exerted a synergistic toxic effect, with significantly lower cell viability than the single treatment groups. Exogenous NMN supplementation completely reversed the FK866-induced reduction in cell viability, but exerted no alleviating effect on DON-induced cytotoxicity. Meanwhile, exogenous NMN failed to effectively increase NAD^+^ levels in untreated HEK293T cells (Figure 3D), and only increased intracellular NAD^+^ levels in DON-treated cells at an extremely high concentration (500 μM) (Figure 3E). These results clearly demonstrate that DON-induced NAD^+^ depletion and cellular damage are not significantly associated with the inhibition of the NAMPT-mediated NAD^+^ salvage synthesis pathway.

### 2.4. DON Induces Impaired Cell Viability by Activating PARP1 to Accelerate NAD^+^ Consumption

The results from Section 2.1, Section 2.2 and Section 2.3 confirmed that although DON reduces intracellular NAD^+^ content, this effect is not mediated by inhibiting the NAD^+^ synthesis pathway. Based on these findings, we hypothesized that DON may disrupt NAD^+^ homeostasis by activating the NAD^+^ consumption pathway to accelerate NAD^+^ depletion. The PARP family, particularly PARP1, is the largest known intracellular consumer of NAD^+^ [22]. PARP1 is markedly activated when cells suffer DNA damage, which leads to rapid NAD^+^ consumption and impaired cell survival and functional homeostasis [44]. Previous studies have found that DON can induce oxidative stress and DNA damage, which are key triggers for PARP1 activation [18]. Therefore, we postulated that DON may trigger PARP1 activation, which consequently accelerates intracellular NAD^+^ consumption, leading to progressive NAD^+^ depletion and eventual cellular injury.

**Figure 4:**
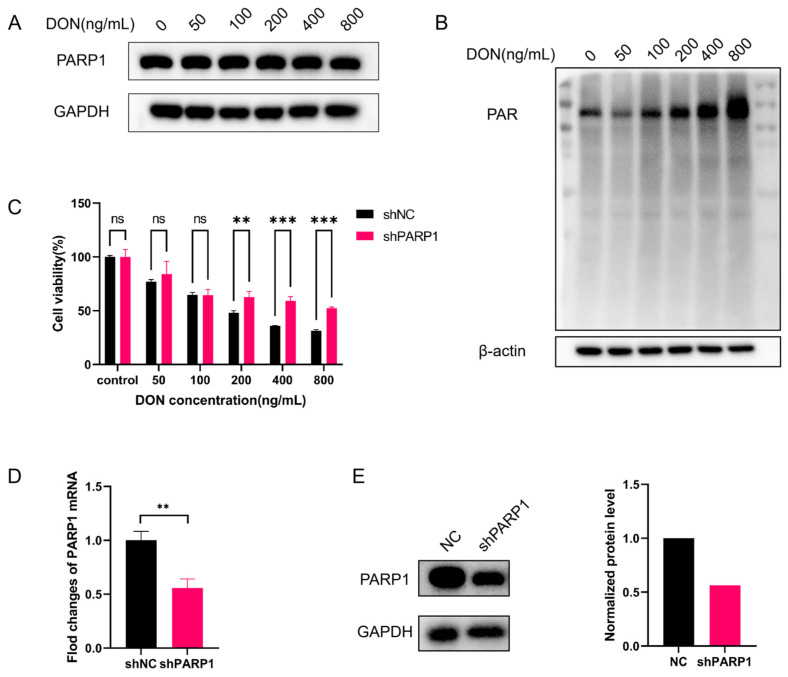
(**A**) PARP1 protein expression in wild-type HEK293T cells treated with gradient DON concentrations for 24 h, analyzed by Western blot (GAPDH as loading control). (**B**) Intracellular poly(ADP-ribose) (PAR) levels in HEK293T cells treated with gradient DON concentrations for 24 h, reflecting PARP1 enzymatic activity. (**C**) Cell viability of sh*PARP1* and negative control (shNC) HEK293T cells after DON treatment for 24 h, detected by CCK-8 assay; the protective effect of *PARP1* knockdown was enhanced with increasing DON concentration. (**D**) Knockdown of *PARP1* was confirmed by qRT-PCR. (**E**) Knockdown of *PARP1* was confirmed by Western blot. Matching empty shRNA vector was used as the negative control (NC). Statistical significance was designated as ** *p* < 0.01, and *** *p* < 0.001; ns = no significant difference.

To verify this hypothesis, we first examined the effect of DON on PARP1 protein expression via Western blot. The results (Figure 4A) showed that PARP1 protein expression did not change significantly in wild-type cells after treatment with gradient concentrations of DON, indicating that DON does not regulate PARP1 protein expression. The level of poly(ADP-ribose) (PAR), the catalytic product of PARP1, directly reflects the enzymatic activity of PARP1 [44]. Therefore, we further measured intracellular PAR levels, and the results (Figure 4B) showed that DON significantly increased intracellular PAR levels in a dose-dependent manner, confirming that DON activates the enzymatic activity of PARP1 rather than upregulating its protein expression.

To clarify the causal link between PARP1 activation and DON-induced cytotoxicity, we measured the viability of sh*PARP1* cell lines (Figure 4E) after DON treatment using the CCK-8 assay (Figure 4C). Compared with the negative control group (shNC group), *PARP1* knockdown significantly alleviated DON-induced reduction in cell viability, and this protective effect was enhanced with increasing DON concentration. Taken together, these results confirmed that DON induced impairment of cell viability by activating PARP1 and accelerating intracellular NAD^+^ consumption, and that PARP1 hyperactivation was the core regulatory event driving DON-mediated cellular damage.

### 2.5. NAM Antagonizes DON-Induced Cytotoxicity by Inhibiting PARP1 Hyperactivation

As a product of the PARP1-catalyzed NAD^+^ hydrolysis reaction, NAM can directly inhibit the catalytic activity of PARP1. Meanwhile, as the primary precursor of NAD^+^, NAM can replenish intracellular NAD^+^ pools [45]. Combined with the results from Section 2.4, this study further investigated whether NAM can act as an antagonist of DON toxicity and the core mechanism underlying its protective effect.

**Figure 5:**
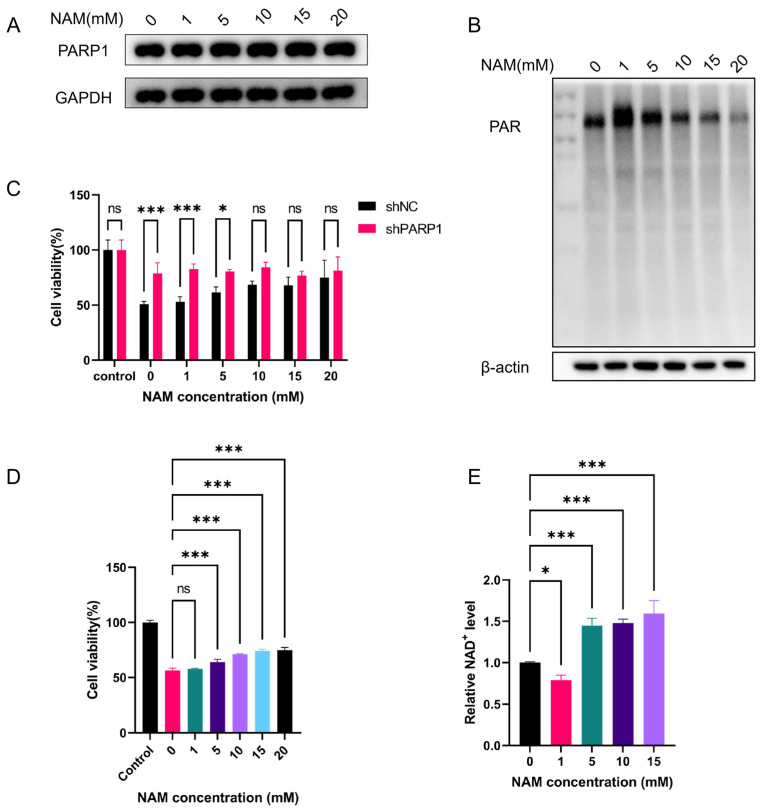
(**A**) PARP1 protein expression in HEK293T cells treated with gradient concentrations of NAM (0, 1, 5, 10, 15, 20 mM) for 24 h, analyzed by Western blot (GAPDH as loading control). (**B**) Intracellular PAR levels in DON-treated (200 ng/mL, 24 h) HEK293T cells treated with gradient NAM concentrations for 24 h. (**C**) Cell viability of sh*PARP1* and shNC HEK293T cells after co-treatment with DON (200 ng/mL) and NAM (0, 1, 5, 10, 15, 20 mM) for 24 h. (**D**) Analysis of the protective effect of gradient NAM concentrations on DON-induced HEK293T cell viability reduction. (**E**) Intracellular NAD^+^ levels in DON-treated HEK293T cells treated with gradient NAM concentrations for 24 h. Statistical significance was designated as * *p* < 0.05, and *** *p* < 0.001; ns = no significant difference.

We first examined the effect of gradient concentrations of NAM on PARP1 protein expression in HEK293T cells by Western blot. The results (Figure 5A) showed that there was no significant change in PARP1 protein expression after treatment with NAM. Further measurement of intracellular PAR levels revealed (Figure 5B) that NAM reduced PAR levels in DON-treated HEK293T cells in a dose-dependent manner, confirming that NAM effectively inhibits DON-induced PARP1 hyperactivation.

**Figure A1:**
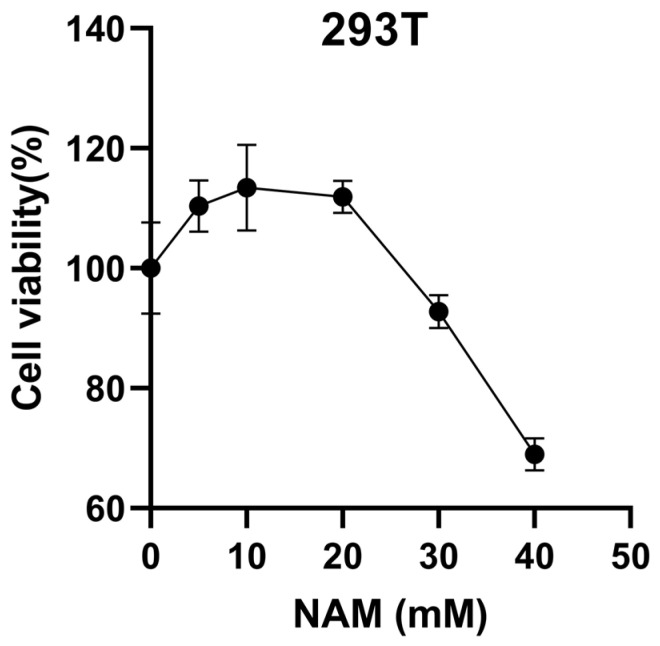
Effect of gradient concentrations of NAM on the viability of HEK293T cells. HEK293T cells were treated with NAM at concentrations of 0, 5, 10, 20, 30 and 40 mM, respectively, detected by CCK-8 assay.

The CCK-8 assay results (Figure 5D) showed that NAM alleviated DON-induced reduction in cell viability in a concentration-dependent manner. Among the treatments evaluated, NAM at concentrations of 10 mM (Figure A1) and above exerted a protective effect comparable to that of *PARP1* knockdown. Notably, NAM treatment failed to further alleviate DON-induced cytotoxicity in the sh*PARP1* cell line (Figure 5C). This result strongly demonstrates that the protective effect of NAM against DON-induced cellular damage is dependent on PARP1. Correspondingly, quantification of intracellular NAD^+^ levels (Figure 5E) showed that NAM supplementation dose-dependently restored NAD^+^ levels in DON-treated cells, which mirrored the recovery pattern of cell viability.

These results confirm that the core mechanism underlying the protective effect of NAM against DON-induced cellular damage is to reduce NAD^+^ consumption by inhibiting the excessive activation of PARP1, while simultaneously replenishing intracellular NAD^+^ depletion caused by DON toxicity as a precursor of NAD^+^, ultimately maintaining intracellular NAD^+^ homeostasis and antagonizing the cytotoxicity of DON.

## 3. Discussion

DON is one of the most prevalent mycotoxins contaminating global food and feed supplies, posing a persistent threat to agricultural sustainability, livestock productivity, and public health [1,3]. As the primary organ responsible for DON excretion, the kidney is highly susceptible to DON-induced injury; yet the molecular mechanisms underlying DON-induced renal damage remain incompletely elucidated, with few targeted mitigation strategies reported to date [12]. NAM, a vitamin B3 derivative and core NAD^+^ precursor, exhibits pleiotropic regulatory effects on energy metabolism, DNA damage repair, and redox homeostasis via modulating NAD^+^-dependent enzymes including PARPs [36,44,45,46]. In this study, we used HEK293T cells as an in vitro model of renal epithelial cells, and demonstrated for the first time that NAM markedly ameliorates DON-induced cytotoxicity by suppressing PARP1 hyperactivation and restoring NAD^+^ homeostasis. Our findings not only delineate the critical role of NAD^+^ homeostasis dysregulation in DON-induced renal toxicity, but also provide a novel and safe intervention strategy for controlling DON hazards in food and feed production.

In this study, we found that DON exposure significantly decreased NAD^+^ levels in porcine serum and HEK293T cells, and exogenous NAD^+^ supplementation alleviated the DON-induced reduction in cell viability in a concentration-dependent manner. Given the central role of NAD^+^ in multiple core biological processes including cellular metabolism, DNA repair, and cell signal transduction, we hypothesized that DON exerts its toxic effects by depleting intracellular and systemic NAD^+^ pools [22,30,34]. To test this hypothesis, we screened for DON-interacting proteins involved in NAD^+^ synthesis via protein microarray, and identified that NAMPT, the rate-limiting enzyme in the NAD^+^ salvage synthesis pathway, specifically binds to DON in vitro. Molecular docking simulation revealed that the primary binding sites were Arg-196 and Asp-219, which form the active site of NAMPT responsible for binding its substrates NAM and PRPP [43]. This binding mode suggests that DON may directly impair the catalytic function of NAMPT by competitively occupying its active site. As the rate-limiting enzyme of the NAD^+^ salvage synthesis pathway, inhibition of NAMPT function would theoretically lead to a decrease in intracellular NAD^+^ levels, which is consistent with the significant reduction in intracellular NAD^+^ levels after DON treatment observed in this study [34].

Further experiments showed that DON regulated NAMPT expression in a biphasic, concentration-dependent manner: it upregulated NAMPT expression at low concentrations and downregulated its expression at high concentrations. However, contrary to our initial hypothesis, we found that *NAMPT* knockdown alleviated DON-induced cellular damage, while *NAMPT* overexpression slightly enhanced the sensitivity of HEK293T cells to DON. This seemingly paradoxical phenomenon can be explained by two aspects: first, *NAMPT* knockdown reduces the conversion of NAM to NMN, leading to intracellular accumulation of NAM, which then competitively inhibits PARP1 activity by occupying its NAD^+^-binding pocket and thus alleviates DON-induced cytotoxicity; second, *NAMPT* overexpression accelerates the consumption of intracellular NAM, weakening its endogenous inhibitory effect on PARP1, and thus exacerbating DON-induced PARP1 hyperactivation and NAD^+^ depletion [47]. This finding further confirms that NAMPT-mediated NAD^+^ synthesis is not the core pathway driving DON-induced renal cell injury, despite the direct binding and enzymatic inhibition of NAMPT by DON.

In vitro enzyme activity assays further confirmed that DON treatment significantly inhibited the enzymatic activity of NAMPT. However, exogenous supplementation with NMN (the catalytic product of NAMPT and direct precursor of NAD^+^) failed to exert a protective effect against DON toxicity, indicating that DON-induced inhibition of NAMPT enzymatic activity is not the main cause of intracellular NAD^+^ depletion. To validate this inference, we further treated HEK293T cells with the specific NAMPT inhibitor FK866 in combination with DON. We found that combined treatment with DON and FK866 exerted a synergistic toxic effect, and exogenous NMN supplementation completely reversed FK866-induced cytotoxicity, but had no alleviating effect on DON-induced damage. In addition, exogenous NMN only increased NAD^+^ levels in DON-treated cells at an extremely high concentration. All these results demonstrate that DON-induced NAD^+^ depletion and cellular damage are not mainly mediated by the blockade of the NAMPT-dependent NAD^+^ salvage synthesis pathway.

Our previous study found that knockdown of the NAD^+^-consuming enzyme *SIRT1* had no significant effect on DON-induced cytotoxicity, whereas treatment with the SIRT1 activator STR1720 increased cellular sensitivity to DON [48]. We speculate that this phenotype may be related to excessive NAD^+^ consumption caused by SIRT1 activation [46]. Based on this, we hypothesized that DON may induce intracellular NAD^+^ depletion, energy metabolism dysregulation, and subsequent cellular damage by activating NAD^+^-consuming enzymes [21]. After entering cells, DON induces massive ROS production, triggers oxidative stress, and causes DNA damage. The PARP family, particularly PARP1, is the largest known intracellular consumer of NAD^+^, and its activity is robustly activated upon DNA damage, leading to rapid NAD^+^ consumption and impaired cell survival and functional homeostasis [49]. Therefore, we speculated that DON may induce intracellular NAD^+^ depletion and subsequent cellular damage by activating PARP1 to accelerate NAD^+^ consumption.

Our experimental data revealed that DON treatment had no effect on cellular PARP1 protein expression, but drove a dose-dependent elevation in the accumulation of PAR, the catalytic product of PARP1-mediated NAD^+^ consumption [37,38]. This finding demonstrates that DON potentiates PARP1 enzymatic activity, rather than promoting its translational upregulation. *PARP1* knockdown significantly alleviated DON-induced reduction in cell viability, and this protective effect was augmented with increasing DON concentration. Taken together, we conclude that DON induces NAD^+^ depletion and subsequent cellular damage by driving PARP1 hyperactivation.

Based on these findings, we proposed a hypothesis: a molecule that can simultaneously inhibit PARP1 activity and reverse intracellular NAD^+^ depletion can effectively antagonize DON-induced toxicity. As a catalytic product of PARP1-mediated NAD^+^ hydrolysis, NAM can directly inhibit the enzymatic activity of PARP1 by competitively occupying its NAD^+^-binding pocket. Meanwhile, as a key precursor for NAD^+^ biosynthesis, NAM can effectively replenish intracellular NAD^+^ pools [37,38]. In addition, NAM is a widely used food additive and dietary supplement with well-validated biosafety, which lays a solid foundation for its agricultural application [50,51]. Our experimental results showed that NAM did not affect PARP1 protein expression, but dose-dependently reduced PAR levels in DON-treated cells, confirming that NAM can effectively inhibit DON-induced PARP1 hyperactivation. Meanwhile, exogenous NAM supplementation effectively restored NAD^+^ levels in DON-treated HEK293T cells. Furthermore, NAM alleviated DON-induced cellular damage in a concentration-dependent manner, while this protective effect was significantly weakened or even abolished in *PARP1* knockdown cells. These results confirm that NAM exerts a robust protective effect against DON-induced cellular damage, and this effect is fully dependent on its inhibition of PARP1 activity. Given the favorable bioavailability of NAM [52], our in vitro mechanistic findings suggest that NAM may have potential as a candidate intervention for DON-induced toxicity. However, it is critical to emphasize that these results are limited to cell culture experiments, and no conclusions can be drawn regarding the in vivo efficacy of NAM supplementation in humans or animals at this stage.

Although this study has systematically elucidated the core mechanism by which nicotinamide ameliorates DON-induced renal cell injury via inhibiting PARP1 hyperactivation and restoring NAD^+^ homeostasis, certain limitations remain. Specifically, this study did not perform in vivo intervention studies using NAM or quantify NAD^+^ concentrations in renal tissue specimens to validate its protective efficacy against DON-induced renal injury and damage to other major target organs, which would have allowed us to further corroborate and extend our current findings.

In conclusion, the findings of this study establish that DON-induced cytotoxicity in HEK293T cells is driven not by inhibition of the NAMPT-mediated NAD^+^ salvage synthesis pathway, but predominantly by aberrant hyperactivation of PARP1, which triggers massive intracellular NAD^+^ depletion and subsequent cellular dysfunction. NAM significantly mitigates DON-induced renal cell damage by suppressing PARP1 hyperactivation and preserving intracellular NAD^+^ homeostasis. This study delineates the molecular mechanism of DON-induced renal cell injury from the perspective of NAD^+^ metabolism, and provides solid in vitro experimental evidence for the development of NAM as a promising candidate for mitigating DON-related toxic hazards in food and feed safety.

## 4. Materials and Methods

### 4.1. Cell Culture and Experimental Reagents

Human embryonic kidney 293T (HEK293T) cells used in this study were sourced from the American Type Culture Collection (ATCC). Routine cell culture was performed in Dulbecco’s Modified Eagle Medium (DMEM, Thermo Fisher Scientific, Waltham, MA, USA) supplemented with 10% (*v*/*v*) fetal bovine serum (FBS, Biological Industries, Kibbutz Beit Haemek, Israel). Cells were incubated in a humidified atmosphere with 5% CO_2_ at a constant 37 °C, and subcultured every 2 to 3 days when cell confluence reached 80–90%.

### 4.2. In Vivo Animal Experimental Protocol

A total of 12 clinically healthy weaned piglets (Duroc × Landrace × Yorkshire three-way crossbred, 28 days old, with uniform initial body weight) were randomly divided into two independent experimental groups (*n* = 6 piglets per group): a blank control group and a DON-challenged treatment group. The control group received a standard basal diet throughout the entire 28-day experimental period, while piglets in the DON challenge group were given the identical basal diet fortified with 2 mg/kg deoxynivalenol (DON) for the same feeding duration. All piglets were individually housed in dedicated metabolic cages, with ad libitum access to clean drinking water and experimental feed throughout the trial. The housing environment was controlled at a constant temperature of 25 ± 2 °C, with a strict 12 h light/12 h dark photoperiod. Upon completion of the feeding trial, whole blood samples were collected from each piglet via anterior vena cava puncture. Serum samples were separated from whole blood via centrifugation at 3000× *g* for 15 min under 4 °C conditions, then immediately snap-frozen and stored at −80 °C pending subsequent analysis of nicotinamide adenine dinucleotide (NAD^+^) content. All animal-related experimental protocols were performed in strict accordance with the Guide for the Care and Use of Laboratory Animals, and were reviewed and approved by the Institutional Animal Care and Use Committee (IACUC) of South China Agricultural University (Ethics Approval Permit No. 2024F222). The complete ethical approval documentation can be provided upon reasonable request.

### 4.3. Cell Viability and Cytotoxicity Evaluation via CCK-8 Assay

Cell viability and cytotoxicity of DON on HEK293T cells were evaluated using the Cell Counting Kit-8 (CCK-8) assay, with reagents sourced from Yeasen Biotech Co., Ltd. (Shanghai, China). Briefly, HEK293T cells were inoculated into 96-well culture plates at a seeding density of 1 × 10^4^ cells per well, and cultured under standard incubation conditions until the cell monolayer reached ~60% confluence. The IC_50_ of DON in HEK293T cells was 207 ng/mL. Thus, 200 ng/mL (corresponding to ~50% cell viability) was chosen as the standard concentration for evaluating intervention agent efficacy. After the designated 24 h drug intervention, 10 μL of CCK-8 working reagent was added to each well, and the plates were subsequently incubated for 1 h at 37 °C in the dark. Wells treated with 0.01% dimethyl sulfoxide (DMSO) were included as the negative control in each assay plate. Following incubation, the optical density (OD) value at 450 nm was quantified using a SpectraMax i3x microplate reader (Molecular Devices, Sunnyvale, CA, USA). All assays were conducted in three independent biological repeats, with six technical replicates set for each treatment condition in every independent experiment. The following compounds were procured from Sigma-Aldrich (St. Louis, MO, USA) and used for cellular interventions: deoxynivalenol (DON), nicotinamide adenine dinucleotide (NAD^+^), nicotinamide (NAM), nicotinamide mononucleotide (NMN), and FK866 (all with a minimum purity of 98%).

### 4.4. Detection of NAD^+^ Content

We performed the 600MRM analysis (Biotree, Shanghai, China) with LC-MS/MS. Serum samples were thawed on ice, vortexed for 30 s, and 100 μL aliquots were extracted with 200 μL water and 1200 μL LC-MS grade acetonitrile–methanol (1:1, *v*/*v*, containing stable isotope-labeled internal standards). After 30 s vortexing, 15 min ice-bath sonication, and 2 h −40 °C incubation, samples were centrifuged at 12,000× *g*, 4 °C for 15 min. Then, 1200 μL supernatant was vacuum-dried via centrifugal concentration, reconstituted in 120 μL 60% acetonitrile, and centrifuged again under identical conditions. Then, 60–70 μL of the final supernatant was transferred to glass vials for LC-MS/MS analysis. UPLC separation was performed on a Waters H-Class system with a Waters Atlantis Premier BEH Z-HILIC Column (1.7 µm, 2.1 mm × 150 mm), using mobile phases A (H_2_O:acetonitrile 8:2, *v*/*v*, 10 mmol/L ammonium acetate) and B (H_2_O:acetonitrile 1:9, *v*/*v*, 10 mmol/L ammonium acetate), both adjusted to pH 9 with aqueous ammonia, at an 8 °C autosampler temperature and a 1 μL injection volume; mass spectrometry was conducted on an AB Sciex QTrap 6500 Plus (AB Sciex LLC, Framingham, MA, USA) (IonSpray Voltage +5000 V/−4500 V, Curtain Gas 35 psi, source temperature 500 °C, Ion Source Gas 1 and 2 both 50 psi), with raw data processed via SCIEX Analyst WorkStation Software (v1.7.2) and metabolite quantification via Data Driven Flow (v1.0.1).

The intracellular NAD^+^ levels were measured using an NAD^+^/NADH assay kit (S0175, WST-8 method, Beyotime, Shanghai, China). The cells were collected and processed using the buffer provided in the kit, and absorbance at 450 nm was measured using a microplate reader. NAD^+^ concentration was calculated according to the standard curve, and normalized to the total protein concentration of each sample to eliminate the differences in cell number among groups.

### 4.5. Protein Microarray Assay

The human protein microarray (containing more than 20,000 human recombinant proteins) was purchased from CDI Laboratories (Baltimore, MD, USA). Cyanine 3-labeled DON (10 μM) was incubated with the protein microarray at 4 °C for 8 h, after which the microarray was washed three times with wash buffer (PBS containing 0.1% Tween-20) to remove unbound DON. Fluorescence signals were scanned using a laser scanner (GenePix 4200A, Molecular Devices, San Jose, CA, USA), and the data were analyzed with GenePix Pro 6.0 software. Proteins with a fluorescence signal intensity ≥1.2-fold above the background were defined as DON-interacting proteins.

### 4.6. Thermal Shift Assay (TSA)

Purified recombinant human NAMPT protein (1 μg/μL, 1.5 mL) was incubated with 100 μM DON or an equal volume of DMSO (solvent control) at 4 °C for 1.5 h. Each incubation mixture was then divided into 6 equal aliquots and heated at a temperature gradient (41.7, 44.7, 49.4, 55.0, 59.7, 63.0 °C) for 3 min, followed by immediate cooling on ice for 5 min. After centrifugation at 12,000× *g* for 10 min at 4 °C to remove precipitated denatured protein, the soluble protein fraction in the supernatant was collected. All samples from the DON treatment group, DMSO control group, and an original unprocessed recombinant NAMPT protein sample (used as the internal standard for normalization and calculation of relative Band Intensity) were subsequently analyzed by Western blot.

### 4.7. Molecular Docking Simulation

The three-dimensional crystal structure of human NAMPT (PDB ID: 4KFN) was retrieved from the Protein Data Bank (PDB, https://www.rcsb.org/), and the chemical structure of DON was obtained from the PubChem database (https://pubchem.ncbi.nlm.nih.gov/, accessed on 11 January 2024). Molecular docking was performed using AutoDock Vina 1.1.2 software. The NAMPT protein structure was preprocessed by removing water molecules and adding hydrogen atoms, and the docking grid was set with the active site of NAMPT as the center (grid box size: 30 × 30 × 30 Å). The docking results were visualized and analyzed using PyMOL 2.5 software.

### 4.8. Quantitative Real-Time PCR (qRT-PCR) for Gene Expression Profiling

**Figure A2:**
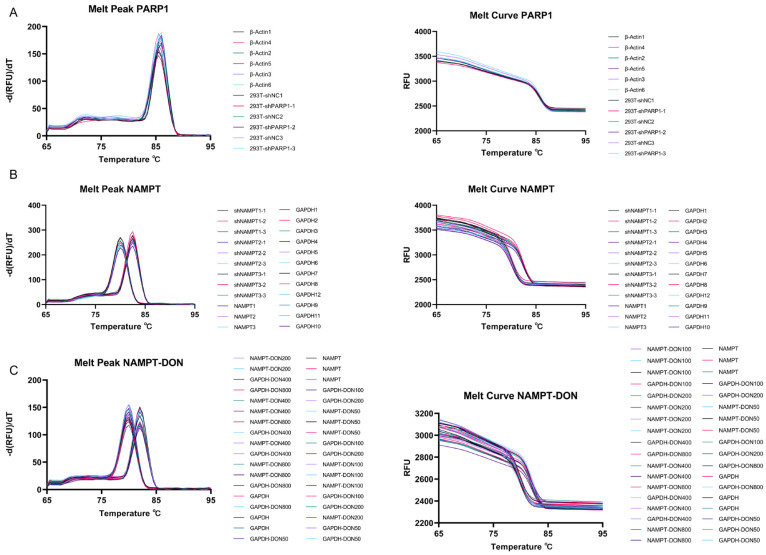
Melting curve analysis of quantitative real-time PCR (qRT-PCR) amplification products to verify amplification specificity. (**A**) Melting peaks (left) and melting curves (right) of Figure 4D. (**B**) Melting peaks (left) and melting curves (right) of Figure 2E. (**C**) Melting peaks (left) and melting curves (right) of Figure 2A. All samples showed single, sharp melting peaks with no detectable primer dimers or non-specific amplification products, confirming the high specificity of the qRT-PCR primers used in this study.

Total RNA was isolated from harvested cell pellets using TRIzol reagent (Life Technologies, Carlsbad, CA, USA) strictly following the manufacturer’s standard protocol. After preliminary assessment of RNA purity (OD260/280 ratio between 1.8 and 2.1) and concentration, 1 μg of qualified total RNA was used per reaction for reverse transcription to synthesize complementary DNA (cDNA) with the ReverTra Ace qPCR RT Master Mix (Toyobo Co., Ltd., Tokyo, Japan). Subsequent qRT-PCR amplification was carried out using the Hieff qPCR SYBR Green Master Mix (Yeasen Biotech Co., Ltd., Shanghai, China) on a real-time fluorescence quantitative PCR system. The GAPDH gene was employed as the endogenous reference for normalization of *NAMPT* expression levels, while β-actin was used as the endogenous reference for normalization of *PARP1* expression levels. The relative transcript abundance of each target gene was calculated using the comparative 2^−ΔΔCt^ method. All primer sequences designed and utilized for qRT-PCR assays are provided in Table A2. The thermal cycling program for amplification was set as an initial pre-denaturation step at 95 °C for 5 min, followed by 40 consecutive cycles of denaturation at 95 °C for 10 s, primer annealing at 57 °C for 20 s, and fragment extension at 72 °C for 20 s. Upon completion of the amplification cycles, melting curve analysis was performed to validate the specificity of each PCR product and rule out non-specific amplification or primer dimer formation. Melting curve analyses for all qRT-PCR assays performed in this study are presented in Figure A2, which confirms the absence of non-specific amplification and primer dimers across all experimental groups.

### 4.9. Western Blotting for Protein Expression Detection

Total cellular protein was extracted using ice-cold RIPA lysis buffer (50 mM Tris-HCl, pH 7.8, 150 mM NaCl, 1% Triton X-100) freshly supplemented with protease and phosphatase inhibitor cocktails (Biomake, Houston, TX, USA). Protein concentrations were determined using a BCA assay kit. Equal amounts of protein (10 μL per well) were separated by 10% SDS-PAGE and electro-transferred onto 0.45 μm PVDF membranes (MilliporeSigma, Boston, MA, USA). Following electrotransfer, membranes were trimmed immediately based on the pre-stained protein molecular weight marker and the predicted molecular masses of the target proteins. Only the membrane regions corresponding to the expected molecular weights of the target proteins were retained for subsequent antibody incubation. Membranes were blocked with 5% (*w*/*v*) non-fat dry milk in TBST for 1 h at room temperature, washed three times with TBST, and incubated overnight at 4 °C with the following primary antibodies diluted in 5% BSA-TBST:

- NAMPT (1:1000, Cat. No. 66385-1-Ig, Proteintech Group, Inc., Wuhan, China);
- PARP1 (1:1000, Cat. No. F2623, Selleck Chemicals, Houston, TX, USA);
- PAR (1:1000, Cat. No. 89190, Cell Signaling Technology, Inc., Danvers, MA, USA);
- β-actin (1:1000, Cat. No. 66009-1-Ig, Proteintech Group, Inc., Wuhan, China);
- GAPDH (1:1000, Cat. No. sc-32233, Santa Cruz Biotechnology, Inc., Dallas, TX, USA).

After primary antibody incubation, membranes were washed three times with TBST and incubated with HRP-conjugated secondary antibody (1:5000, Cat. No. 18665S, Cell Signaling Technology) for 1 h at room temperature. Protein bands were visualized using Beyo ECL Star chemiluminescence kit (Beyotime Biotechnology, Shanghai, China) and captured with a ChemiDoc High-Sensitivity Molecular Imaging System (Bio-Rad Laboratories, Hercules, CA, USA).

### 4.10. In Vitro NAMPT Enzyme Activity Assay

Three experimental groups were established: the negative control (NC) group, the toxin treatment group, and the blank group. In the blank group, DON was replaced with an equal volume of DMSO. First, 27 μL of reaction mixture containing Tris-HCl (50 mM), MgCl_2_ (10 mM), ATP (2 mM), DTT (2 mM), NAMPT (30 nM) and DON (3 μM) was prepared in each microcentrifuge tube and pre-incubated at 37 °C for 5 min. Then, 56.3 μL of premixed substrate solution containing NAM (25 μM) and PRPP (50 μM) was added, mixed thoroughly, and the mixture was immediately incubated at 37 °C for 10 min. The enzymatic reaction was terminated by heating at 100 °C for 1 min in a metal bath. Subsequently, the tubes were placed on ice, and 90 μL of 20% acetophenone followed by 90 μL of 2 M KOH were sequentially added and mixed thoroughly, followed by incubation on ice for 10 min. Next, 405 μL of 88% formic acid was added, mixed well, and incubated at 37 °C for 30 min. Finally, 100 μL of each sample was transferred to a 96-well plate with 7 technical replicates per sample. Fluorescence intensity was measured using a microplate reader in fluorescence mode at an excitation wavelength of 382 nm and an emission wavelength of 445 nm. Relative enzyme activity was calculated using the formula: Enzyme activity (E%) = (Toxin treatment group − Blank group)/(Control group − Blank group).

### 4.11. Construction of NAMPT Recombinant Plasmid

Primers targeting the human *NAMPT* coding sequence (GenBank accession No. NM_005746.3) were designed and synthesized by Sangon Biotech (Shanghai, China) (Table A3). The target fragment was amplified from HEK293T cell cDNA library via PCR using Phanta Max Super-Fidelity DNA Polymerase (Vazyme Biotech, Nanjing, China), with cycling parameters: 95 °C for 45 s; 35 cycles of 95 °C 15 s, 56 °C 15 s, 72 °C 90 s; 72 °C 5 min final extension. PCR products were gel-purified. pET-28a vector was linearized via NcoI/EcoRI double digestion (ABclonal, Wuhan, Hubei Province, China) at 37 °C for 60 min and gel-purified. Homologous recombination between the *NAMPT* insert and linearized vector was performed using 2 × Assembly Mix at 50 °C for 20 min. Recombinant plasmids were transformed into *E. coli* DH5α competent cells (Tiangen Biotech, Beijing, China) via standard heat shock, screened on LB plates with 50 μg/mL kanamycin, verified by Sanger sequencing, and extracted using a commercial plasmid kit (CWBIO, Taizhou, China).

### 4.12. Expression and Purification of Recombinant NAMPT

Validated pET-28a-*NAMPT* plasmid was transformed into *E. coli* BL21 (DE3) competent cells (Tiangen Biotech) for protein expression. Single colonies were activated in kanamycin-containing LB medium at 37 °C, 200 rpm for 6 h, then inoculated 1:100 (*v*/*v*) into 500 mL LB medium and cultured to OD_600_ = 0.6. Protein expression was induced with 1 mM IPTG at 25 °C, 150 rpm for 24 h. Bacterial cells were harvested by centrifugation at 3500× *g*, 4 °C for 20 min, resuspended in ice-cold Buffer A (20 mM Tris-HCl, 500 mM NaCl, 20 mM imidazole, pH 8.0) supplemented with 1 mM PMSF, and lysed via sonication. The lysate was centrifuged at 12,000× *g*, 4 °C for 30 min, and the supernatant was filtered through a 0.45 μm membrane. Then, 6 × His-tagged recombinant NAMPT was purified via Ni-NTA affinity chromatography on an ÄKTA Pure system. After equilibration with Buffer A, the sample was loaded at 1 mL/min, followed by gradient elution with Buffer B (20 mM Tris-HCl, 500 mM NaCl, 500 mM imidazole, pH 8.0) at a constant 1 mL/min flow rate. High-purity eluted fractions were pooled for subsequent experiments.

### 4.13. Construction of NAMPT Vector

shRNA targeting human *NAMPT* was designed via the Merck shRNA database, with oligonucleotides synthesized by Sangon Biotech (Table A3). Sense and antisense oligonucleotides were annealed to generate double-stranded shRNA inserts. The pLKO.1 vector was linearized via BshT I/EcoR I double digestion (ABclonal, Wuhan, Hubei Province, China) at 37 °C for 3 h and gel-purified. The shRNA insert was ligated into linearized pLKO.1 using T4 DNA ligase (NEB) at 22 °C overnight. The ligation product was transformed into *E. coli* Stbl3 competent cells (Tiangen Biotech, Beijing, China). Positive clones were verified by colony PCR and Sanger sequencing, and endotoxin-free plasmids were extracted with a commercial kit (CWBIO, Taizhou, China). 

Primers targeting the full-length CDS of human *NAMPT* (GenBank accession No. NM_005746.3) were synthesized by Sangon Biotech (Table A3). The target fragment was amplified from HEK293T cell cDNA via PCR with a high-fidelity DNA polymerase (Vazyme Biotech, Nanjing, China). The pSIN vector was linearized via Eco105I digestion (NEB) at 37 °C for 3 h, and the digested vector and PCR product were purified. Homologous recombination between the linearized vector and *NAMPT* insert was performed using 2× Assembly Mix (Vazyme Biotech) at 50 °C for 20 min. The recombination product was transformed into *E. coli* Stbl3 competent cells, with positive clones verified by colony PCR and Sanger sequencing. Endotoxin-free plasmids were extracted from validated clones for subsequent experiments.

### 4.14. Establishment of Stable NAMPT-Modified Cell Lines

Lentiviruses for *NAMPT* knockdown or overexpression were packaged in HEK293T cells via PEI (Sigma-Aldrich) co-transfection at a 3:3:2 plasmid ratio (target construct: psPAX2: pMD2.G). Medium was replaced 5 h post-transfection; viral supernatants were harvested at 24 h and 48 h, clarified by centrifugation, sterile-filtered (0.45 μm), and stored at −80 °C. Target cells were transduced with lentivirus in medium containing 8 μg/mL polybrene (Sigma-Aldrich), then selected with 2 μg/mL puromycin (Sigma-Aldrich) for 7 days to generate stable *NAMPT*-modified cell lines, which were cryopreserved for subsequent assays.

### 4.15. Statistical Analysis

All statistical analyses in this study were performed using GraphPad Prism 9.0 software (GraphPad Software, Inc., San Diego, CA, USA). Experimental data are presented as the mean ± standard error of the mean (SEM) for each experimental group. For comparisons between two independent groups, the unpaired two-tailed Student *t*-test was adopted for difference analysis. For single-factor experimental designs with more than two treatment groups, one-way analysis of variance (ANOVA) was performed, followed by Tukey’s post hoc test for multiple pairwise comparisons. For two-factor experimental designs, two-way ANOVA was conducted, with Tukey’s post hoc test used for subsequent multiple comparisons between groups. Statistical significance was set at three levels: * *p* < 0.05, ** *p* < 0.01, and *** *p* < 0.001; the notation “ns” was used to indicate no statistically significant difference between the compared groups. All assays were carried out in a minimum of three independent biological replicates to verify the stability and reproducibility of the obtained results.

## Data Availability

The original contributions presented in this study are included in the article. Further inquiries can be directed to the corresponding authors.

## Abbreviations

The following abbreviations are used in this manuscript:

CCK-8	Cell Counting Kit-8
TSA	Thermal shift assay
CHOP	C/EBP homologous protein
DON	Deoxynivalenol
HEK293T	Human embryonic kidney 293T cells
JNK	c-Jun N-terminal kinase
NAD^+^	Nicotinamide adenine dinucleotide
NAM	Nicotinamide
NAMPT	Nicotinamide phosphoribosyltransferase
NMN	Nicotinamide mononucleotide
NMNAT	Nicotinamide mononucleotide adenylyltransferase
PAR	Poly(ADP-ribose)
PARP1	Poly(ADP-ribose) polymerase 1
PRPP	5-phosphoribosyl-1-pyrophosphate
ROS	Reactive oxygen species
TNF-α	Tumor necrosis factor-α
UPR	Unfolded protein response

## Appendix A

**Table A1:** Results of partial protein microarray.

Name	Accession No.	Long Gene Name	SNR-DON-Cy3
NAMPT	NM_005746.3	Nicotinamide phosphoribosyltransferase	1.215
GMPR	NM_006877.4	Guanosine monophosphate reductase	1.253
PRPS1	NM_002764.4	Phosphoribosyl pyrophosphate synthetase 1	1.256
PSMD4	NM_002810.4	Proteasome 26S subunit ubiquitin receptor, non-ATPase 4	1.189
GNMT	NM_018960.6	Glycine N-methyltransferase	1.164

**Table A2:** Primer sequences for qRT-PCR.

Primers	Sequences (5′-3′)
*NAMPT*-F	GCAGAAGCCGAGTTCAACATC
*NAMPT*-R	TTTTCACGGCATTCAAAGTAGGA
*PARP1*-F	TTGGGGAAGCTGAGCAAAAG
*PARP1*-R	TCAAGCATTTCCACCTTGGC

**Table A3:** Primers used for vectors.

Primers	Accession No.	Sequences (5’-3’)
*NAMPT*-His-F	NM_005746.3	AGAAGGAGATATACCATGGATTACAAGGATGACGACGATAAGAATCCTGCGGGCAGAAG
*NAMPT*-His-R		TCGACGGAGCTCGAATTCATGTGGTGATGGTGATGATGATGATGTGCTGCTTCC
sh*NAMPT*-F	NM_005746.3	CCGGGCAGAAGCCGAGTTCAACATCCTCGAG GATGTTGAACTCGGCTTCTGCTTTTTG
sh*NAMPT*-R		AATTCAAAAAGCAGAAGCCGAGTTCAACATCCTCGAGGATGTTGAACTCGGCTTCTGC
OE-*NAMPT*-F	NM_005746.3	GGTGTCGTGAGGATTTACATGAATCCTGCGGCAGAAGCC
OE-*NAMPT*-R		GTCATCCTTGTAATCTACATGATGTGCTGCTTCCAGTTCAAT
sh*PARP1*-F	NM_001618.4	CCGGCGACCTGATCTGGAACATCAACTCGAGTTGATGTTCCAGATCAGGTCGTTTTTG
sh*PARP1*-R		AATTCAAAAACGACCTGATCTGGAACATCAACTCGAGTTGATGTTCCAGATCAGGTCG
